# The Addition of All-Trans Retinoic Acid to Chemotherapy May Not Improve the Outcome of Patient with *NPM1* Mutated Acute Myeloid Leukemia

**DOI:** 10.3389/fonc.2013.00218

**Published:** 2013-09-06

**Authors:** Aziz Nazha, Carlos Bueso-Ramos, Eli Estey, Stefan Faderl, Susan O’Brien, Michael H. Fernandez, Martin Nguyen, Charles Koller, Emil Freireich, Miloslav Beran, Sherry Pierce, Michael Keating, Jorge Cortes, Hagop Kantarjian, Farhad Ravandi

**Affiliations:** ^1^Department of Leukemia, University of Texas, Austin, TX, USA; ^2^Department of Hematopathology, University of Texas, Austin, TX, USA; ^3^MD Anderson Cancer Center, Houston, TX, USA; ^4^Clinical Research Division, Fred Hutchinson Cancer Research Center, Seattle, WA, USA

**Keywords:** *NPM1*, ATRA, AML, elderly, chemotherapy

## Abstract

**Background:** Previous studies have suggested that *NPM1* mutations may be a marker for response to all-trans retinoic acid (ATRA) given as an adjunct to intensive chemotherapy in older patients with acute myeloid leukemia (AML).

**Patients and Methods:** We examined the impact of the addition of ATRA among patients with diploid cytogenetics treated on a randomized phase II study of fludarabine + cytarabine + idarubicine ± G-CSF ± ATRA with available data on their *NPM1* mutation status. Between September 1995 and November 1997, 215 patients were enrolled in the study. Among them, 70 patients had diploid cytogenetic and are the subjects of this analysis.

**Results:** The median age of the 70 patients was 66 years (range 23–87). Twenty (29%) of patients had *NPM1* mutations. Among them 7 (35%) did and 13 (65%) did not receive ATRA in combination with chemotherapy. Complete remission (CR) was achieved in 71% of patients treated with ATRA as compared to 69% without ATRA (*P* = 0.62). With median follow-up of 12.5 years, the overall survival (OS), event-free survival (EFS), and relapse-free survival (RFS) were similar among patients who received ATRA compared to no ATRA regardless of *NPM1* mutation status.

**Conclusion:** The addition of ATRA to intensive chemotherapy did not affect the overall outcome of patients with AML regardless of *NPM1* mutation status.

## Introduction

The addition of all-trans retinoic acid (ATRA) to chemotherapy has changed the natural history of acute promyelocytic leukemia (APL) by inducing terminal differentiation of the promyelocyte into mature cells (1, 2). Preclinical data suggests that ATRA may also sensitize non-APL leukemic blasts to cytarabine and anthracyclines by downregulating bcl-2 and related proteins that inhibit apoptosis (3–6). These observations provided a rationale for several clinical trials to evaluate the impact of adding ATRA to conventional chemotherapy in non-APL patients; however, the reports of these trials have been conflicting. Venditti et al. first reported a significantly higher complete remission (CR) rate when ATRA was added to low dose cytarabine in 33 patients with poor risk AML (7). However, several subsequent trials have failed to show a benefit from adding ATRA to conventional chemotherapies (8–10).

Nucleophosmin (*NPM1*) gene mutations are among the most common gene alteration in acute myeloid leukemia (AML) and mainly occur in AML with normal karyotype (NK-AML) (11–13). *NPM1* plays an important role in cell growth, proliferation, and terminal differentiation (14). The prognostic impact of *NPM1* mutations is favorable among patients with NK-AML (12, 15). However, the presence of concomitant *fms*-like tyrosine kinase 3 internal tandem duplication (*FLT3*-ITD) mutation will worsen the outcome (16).

In a subgroup analysis of their AML HD98B randomized trial in elderly patients with AML, Schlenk et al. showed a higher response rate and better relapse-free survival (RFS) and overall survival (OS) only in patients with *NPM1* mutations without FLT3-ITD (NPM+/*FLT3*−ITD−) who received ATRA as part of their induction regimen (17); however, a subgroup analysis of the MRC AML12 trial failed to show a similar benefit in NPM+/FLT3− patients (18).

Here we evaluate the impact of the addition of ATRA to intensive chemotherapy in patients with NK− AML and *NPM1* mutations.

## Materials and Methods

### Patients

Between September 1995 and November 1997, 215 patients with AML and high risk myelodysplastic syndrome (MDS; blasts 10–19%) were enrolled in a phase II randomized clinical trial of fludarabine, cytarabine, and Idarubicin ± A TRA ± granulocyte colony-stimulating factor (G-CSF) (8). The trial design and results have been reported previously (8). Patients were eligible to participate if they had one of the following features: antecedent hematological disorder (AHD) defined as a history of an abnormal blood count (hemoglobin, ≤12 g/dL, or neutrophils ≤1,500/μL, or white blood cells (WBC) ≤10,000/μL, or platelet count ≤150,000/μL) documented to be present for at least 1 month before their presentation to MD Anderson Cancer Center (MDACC), therapy-related AML or MDS, high bilirubin (>2.9 mg/mL) or creatinine (>1.5 mg/mL). Patients were randomly assigned to receive (1) fludarabine 30 mg/m^2^ × days (1–4) + cytarabine 2 g/m^2^ days (1–4) + idarubicin 12 mg/m^2^ days (2–4) = FAI, (2) FAI + G-CSF (200 mg/m^2^ daily), (3) FAI + ATRA 45 mg/m^2^ in two divided dose, or (4) FAI + ATRA + G-CSF as described previously (8). All patients signed informed consent to participate in the trial in accordance with the guidelines reported in the Declaration of Helsinki. The study was approved by the Institutional Review Board (IRB) at MDACC. Seventy patients with NK-AML who participated in this study and had stored bone marrow biopsy specimens were the subject of this analysis.

### Detection of *NPM1* mutations

Bone marrow specimens were examined by immunohistochemistry (IHC) for the presence of cytoplasmic NPM1 (corresponding to *NPM1* mutations). To detect *NPM1* mutations, exon 12 was amplified by PCR using the following primers: GATGTTGAACTATGCAAAGAGACA (forward) and AACCAAGCAAAGGGTGGAGTT (reverse). The PCR products were purified by MinElute TM PCR purification Kit (QIAGEN, Valencia, CA, USA) and directly sequenced using the GGCATTTTGGACAACACA (reverse) primer (Sanger sequencing) using the fluorescence dye chain-terminator chemistry method on ABI Prism 3700 DNA Analyzer (Applied Biosystems, Foster City, CA, USA), and analyzed by using the 310 Genetic Analyzer (Sanger sequencing; Applied Biosystems).

Immunohistochemistry studies were performed using previously described methods (19). Routinely processed BM trephine biopsy tissue sections were subjected to antigen retrieval and immunostained with an anti-NPM1 monoclonal antibody, clone 376 (Dako, Carpinteria, CA, USA) using an alkaline phosphatase monoclonal anti-alkaline phosphatase (APAAP) technique (20). In addition, the BM tissue sections were stained in parallel with a mouse monoclonal antibody directed against nucleolin (C23) (Santa Cruz, Biotechnology, Santa Cruz, CA, USA), another nucleolar protein, which served as a nuclear staining control. In *NPM1* mutated AML cases, NPM protein is abnormally localized in the cytoplasm of most leukemic cells whereas nucleolin/C23 expression is restricted to the nucleus (19). In *NPM1* unmutated cases, NPM protein is restricted to the nucleus.

We found a complete concordance between the results obtained by IHC and the results obtained by DNA sequencing of *NPM1*. Only two cases showed discordance between results obtained by IHC and DNA sequencing: nuclear NPM1 protein by IHC but DNA sequencing showed *NPM1* mutation.

### Definitions of outcome

Complete remission was defined as less than 5% bone marrow blasts, an absolute neutrophil count of 1.0 × 10^9^/L or more, a platelet count of 100 × 10^9^/L or more, no blasts in the peripheral blood, and no extramedullary leukemia. Failures were defined as either refractory disease or early death (death less than 7 days after completion of the first course of induction therapy). Relapse was defined as more than 5% bone marrow blasts unrelated to recovery from the preceding course of chemotherapy or new extramedullary leukemia in patients with previously documented CR.

Overall survival was measured from the time of randomization to time of death or last follows up. Event-free survival (EFS) was measured form the time of randomization to the date of an event, defined as death, relapse, or failure to achieve CR due to resistant disease. RFS was measured from the date of achievement of CR to the date of death in CR, relapse, or at last follow-up.

### Statistical analysis

Differences among variables were evaluated by the Fisher exact test and Mann–Whitney *U* test for categorical and numerical variables, respectively. Time-to-event analyses were performed by the Kaplan–Meier method, and curves were compared with the two-tailed log rank test. A two sided *P* value <0.05 was considered to be statistically significant. All analyses were performed using Statistica 10 software (StatSoft Inc., Tulsa, OK, USA).

## Results

### Patient characteristics

Among the 70 patients with NK-AML who were included in this analysis, 20 (29%) patients had *NPM1* mutations. Patient’s characteristics are summarized in Table 1. The median age for the entire group was 66 (range, 23–87). Forty two (60%) had a history of AHD. A total of 36 (51%) patients were treated with ATRA in combination with chemotherapy. Patients with *NPM1* mutations have similar clinical characteristics compared to patients with wild type *NMP1* except hemoglobin levels which were lower in patients with wild type *NPM1* (*P* = 0.04). Among them, seven (35%) patients received treatment with ATRA plus chemotherapy.

**Table 1:** **Patient characteristics**.

Characteristics	No. (%)	*NPM1* mut. no. (%)	*NPM1* wt. no. (%)	*P* value
No.	70	20 (29)	50 (71)	
Median age, years (range)	66 (23–87)	64 (40–81)	67 (23–87)	0.58
≤60	23 (33)	7 (35)	16 (32)	0.20
>60	47 (67)	13 (75)	34 (68)	
AHD	42 (60%)	11 (55)	31 (62)	0.16
Median WBC × 10^9^/L (range)	12.8 (0.3–245)	10.7 (0.5–245)	13.7 (0.3–196)	0.13
Median hemoglobin g/dL (range)	7.8 (2.9–12.5)	8.2 (6.9–12.5)	7.8 (2.9–12.2)	0.04
Median platelets × 10^3^/mL (range)	55 (8–334)	55 (12–275)	55 (8–334)	0.41
Median BM blasts % (range)	52 (11–94)	54 (13–91)	52 (11–94)	0.68
Treatment with ATRA				0.11
Yes	36 (51)	7 (35)	29 (58)	
No	34 (49)	13 (65)	21 (42)	

**NPM1*, nucleophosmin 1; wt, wild type; mut, mutated; AHD, antecedent hematologic disorder; WBC, white blood cell count; BM, bone marrow; ATRA, all-trans retinoic acid*.

### Response rate

The CR rate for the entire group was 63%. Patients treated with ATRA in combination with chemotherapy had similar response rate to patients who did not receive ATRA (67 vs. 59%, respectively, *P* = 0.14). The CR rate was similar for patients with and without mutated *NPM1* (70 vs. 60%, respectively, *P* = 0.43). CR rate in patients with *NPM1* mutations did no differ according to whether they received ATRA or not (71 vs. 69%, respectively, *P* = 0.62) (Table 2). Early induction mortality was also similar among patients who received ATRA vs. no ATRA regardless of the *NPM1* mutations status (Table 2).

**Table 2:** **Outcome by *NPM1* mutations status and treatment with ATRA**.

Parameter	*NPM1* wt.	*NPM1* mut.	*P* value
Total no.	50	20	
ATRA	29 (58)	7 (35)	0.07
No ATRA	21 (42)	13 (65)	
Response rate
ATRA	19 (66)	5 (71)	0.08
No ATRA	11 (52)	9 (69)	
4 weeks mortality
ATRA	5 (17)	1 (14)	0.34
No ATRA	4 (18)	3 (23)	
8 weeks mortality
ATRA	8 (28)	1 (14)	0.11
No ATRA	6 (28)	5 (38)	
Median OS (weeks)
ATRA	52 (1–684)	41 (3–452)	0.78
No ATRA	41 (2–360)	60 (1–735)	
Median EFS (weeks)
ATRA	30 (1–684)	34 (3–176)	0.18
No ATRA	18 (2–232)	31 (1–650)	
Median RFS (weeks)
ATRA	41 (15–680)	82 (10–173)	0.21
No ATRA	37 (9–228)	56 (1–646)	

**NPM1*, nucleophosmin; WT, wild type; mut, mutated; ATRA, all-trans retinoic acid; OS, overall survival; EFS, event-free survival; RFS, relapse-free survival*.

### Survival analysis

**Figure 1:**
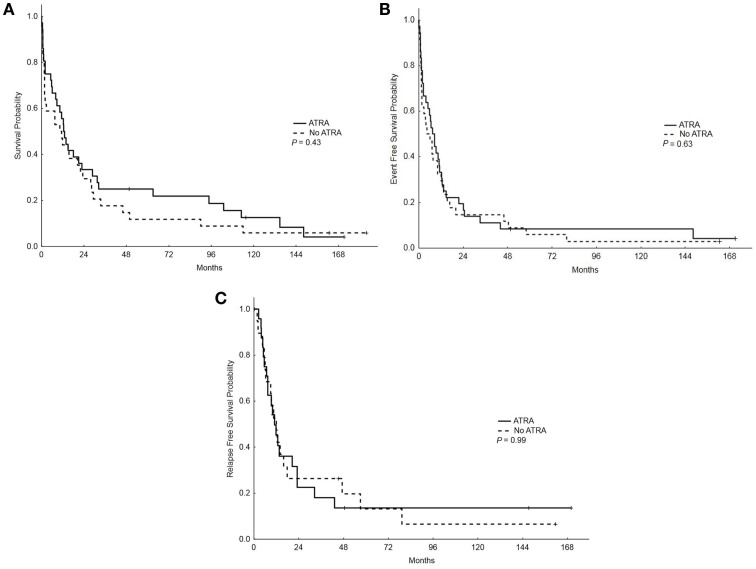
**Overall survival, event-free survival, and relapse-free survival (RFS) in patients who received ATRA vs. no ATRA**. **(A)** Overall survival among patients who received ATRA vs. no ATRA. **(B)** Event-free survival among patients who received ATRA vs. no ATRA. **(C)** RFS among patients who received ATRA vs. no ATRA.

**Figure 2:**
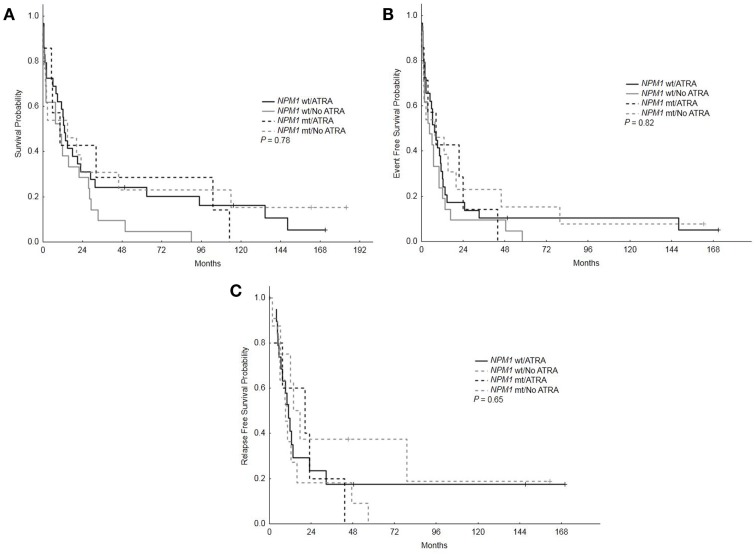
**Overall survival, event-free survival, and relapse-free survival based on *NPM1* mutations status and treatment with ATRA**. **(A)** Overall survival among patients with mutated vs. wild type *NPM1* who received ATRA vs. no ATRA. **(B)** Event-free survival among patients with mutated vs. wild type *NPM1* who received ATRA vs. no ATRA. **(C)** RFS among patients with mutated vs. wild type *NPM1* who received ATRA vs. no ATRA.

With a median follow-up of 12.5 years, the median OS, EFS, and RFS for the entire group were 11.5 months (range, 0.25–183.75), 7 months (range, 0.25–171), and 11.5 months (range, 0.25–170), respectively. There were no differences in the OS, EFS, and RFS among patients who treated with ATRA vs. No ATRA (Table 2; Figure 1). More importantly, the addition of ATRA to induction chemotherapy did not affect the OS, EFS, and RFS of patients with mutated *NPM1* (Table 2; Figure 2).

## Discussion

In this randomized clinical trial of fludarabine, cytarabine, and Idarubicin ± ATRA ± G-CSF, the addition of ATRA to intensive chemotherapy did not improve the outcome (CR, OS, EFS, and RFS) of patients with non-APL AML (8). In addition, in subgroup analysis, patients with diploid karyotype and mutated *NPM1* did not benefit from the addition of ATRA to their chemotherapy and had similar outcome compared to patients with wild type *NPM1*.

Several clinical trials have evaluated the impact of the addition of ATRA to intensive chemotherapy in non-APL AML; however, the results have not been consistent. Venditti et al. reported that the combination of ATRA and low dose cytarabine was associated with a high CR rate in patients with poor risk AML who were unfit to receive intensive chemotherapy (7). However, several subsequent trials have not confirmed this result (8–10). We previously reported a randomized phase II trial of ATRA in combination with fludarabine, cytarabine, and Idarubicin ±G-CSF and found that the addition of ATRA did not impact the outcome among patients with poor risk AML and high risk MDS (8). This result was similar to three large randomized trials conducted by the British Medical Research Council (MRC) in medically unfit patients (MRC AML14) (10), newly diagnosed younger patients (MRC AML12), and in relapsed refractory patients (MRC AML-HR) (9). All of these trials failed to show any significant effect of ATRA in non-APL AML.

In a subgroup analysis of a randomized trial conducted by the German–Austrian AML Study Group (AMLSG), Schlenk et al. showed that the addition of ATRA to intensive chemotherapy was associated with a significant improvement in response rate, OS, and EFS only in elderly patients who were *NPM1*+/*FLT3*−ITD−(17). In contrast, subgroup analysis of MRC AML 12 trial failed to show any benefit from the addition of ATRA to intensive chemotherapy in general and specifically in patients with *NPM1* mutations with or without *FLT3*-ITD mutations in terms of response rate (*P* = 0.6 and 0.1, respectively), relapse (*P* = 0.8 and 0.2, respectively), and OS (*P* = 0.2 and 0.3, respectively) (18).

The differences in the results of these trials may be related to differences in the patient populations as well as the chemotherapy regimens used in combination with ATRA. Furthermore, the dosing schedule of ATRA and whether it is administered before, during, or after exposure to cytotoxic chemotherapies may be important.

Although preclinical data suggests that ATRA may enhance the apoptotic effect of drugs such as cytarabine and daunorubicin through the suppression of bcl-2, the exact mechanisms by which ATRA may affect leukemic myeloid cells with mutant *NPM1* is not fully understood. Preclinical studies have suggested that ATRA may induce apoptosis in *NPM1* mutated AML cell lines by down-regulating NPM1 protein (20).

Limitations of this study include the small sample size of *NPM1* mutated patients and that other mutations such as *FLT3*-ITD were not analyzed in this retrospective study; however, the impact of *FLT3*-ITD status on the response to ATRA in patients with mutated *NPM1* remains controversial (18).

In summary, the addition of ATRA to intensive chemotherapy did not impact the overall outcome of patients with AML. There was no significant difference in CR rate, EFS, or OS in *NPM1* mutated diploid AML receiving ATRA as an adjunct to chemotherapy.

## Conflict of Interest Statement

The authors declare that the research was conducted in the absence of any commercial or financial relationships that could be construed as a potential conflict of interest.
